# Pro-invasive stimuli and the interacting protein Hsp70 favour the route of alpha-enolase to the cell surface

**DOI:** 10.1038/s41598-017-04185-8

**Published:** 2017-06-19

**Authors:** Giovanni Perconti, Cristina Maranto, Daniele P. Romancino, Patrizia Rubino, Salvatore Feo, Antonella Bongiovanni, Agata Giallongo

**Affiliations:** 1 Institute of Biomedicine and Molecular Immunology “A. Monroy” (IBIM), National Research Council (CNR), Palermo, Italy; 2 Department of Biological, Chemical and Pharmaceutical Sciences and Technologies (STEBICEF), University of Palermo, Palermo, Italy

## Abstract

Cell surface expression of alpha-enolase, a glycolytic enzyme displaying moonlighting activities, has been shown to contribute to the motility and invasiveness of cancer cells through the protein non-enzymatic function of binding plasminogen and enhancing plasmin formation. Although a few recent records indicate the involvement of protein partners in the localization of alpha-enolase to the plasma membrane, the cellular mechanisms underlying surface exposure remain largely elusive. Searching for novel interactors and signalling pathways, we used low-metastatic breast cancer cells, a doxorubicin-resistant counterpart and a non-tumourigenic mammary epithelial cell line. Here, we demonstrate by a combination of experimental approaches that epidermal growth factor (EGF) exposure, like lipopolysaccharide (LPS) exposure, promotes the surface expression of alpha-enolase. We also establish Heat shock protein 70 (Hsp70), a multifunctional chaperone distributed in intracellular, plasma membrane and extracellular compartments, as a novel alpha-enolase interactor and demonstrate a functional involvement of Hsp70 in the surface localization of alpha-enolase. Our results contribute to shedding light on the control of surface expression of alpha-enolase in non-tumourigenic and cancer cells and suggest novel targets to counteract the metastatic potential of tumours.

## Introduction

An increasing number of proteins are being identified as multifunctional^1^. Most of these are enzymes, which in addition to their catalytic function are involved in fully unrelated processes, such as the glycolytic enzyme alpha-enolase, which was one of the first moonlighting proteins to be identified^2^. Multiple subcellular localizations characterize alpha-enolase, which functions as a plasminogen receptor when localized on the cell surface, and available data have demonstrated its interaction with plasminogen in prokaryotic and eukaryotic cells^3^. Mammalian tumour cells use the activation of plasminogen in plasmin to invade tissue and form metastases^4^. Recently, researchers have linked both pericellular plasminogen activation and cell surface alpha-enolase to migration and invasion in lung and pancreatic cancer, and these studies have proposed targeting cancer cells with specific anti-alpha-enolase antibodies as a promising approach to suppress tumour metastasis^5, 6^. Due to the large interest in novel therapeutic strategies to counteract cancer spreading, stimuli and signalling pathways that can cooperate to induce the surface localization of alpha-enolase are attractive objects of study.

Increased expression of surface alpha-enolase following LPS exposure was originally reported for the U937 macrophage cell line and human blood monocytes^7^. LPS, a component of the outer membrane of Gram-negative bacteria, exerts its biological effects by binding to Toll-like receptor 4 (TLR4), a recognition receptor of the innate immune system. Some evidence shows that functional TLR4 receptors are expressed on a variety of tumours, including breast cancer, where the silencing of TLR4 results in decreased cellular growth and proliferation, whereas its stimulation promotes tumourigenesis and metastatic lesions^8, 9^. So far, one single report has shown that the LPS triggers the translocation of alpha-enolase to cell surface in breast cancer cell lines and, in addition to LPS, positively correlated this upregulation to other stimuli promoting tumour progression, such as Transforming growth factor (TGF)-β, Tumor necrosis factor (TNF)-α and chemokine ligand 2 (CCL2)^10^.

EGF-induced signalling is also commonly associated with tumour growth, and the overexpression of EGF receptor (EGFR) family members is reported in many human tumours, including lung, colon and breast carcinoma^11^. It is well documented that EGFR activation induces cancer cell migration and invasion, promoting epithelial-mesenchymal transition (EMT) and metastasis^12^; however, to date, no association between the pro-invasive function of EGF and the increased surface expression of alpha-enolase has been reported.

Despite the existence of several reports indicating the active role of surface alpha-enolase in driving cancer cell invasion and metastasis formation, the molecular mechanisms underlying its transport from cytoplasm to cell membrane are still the object of hypotheses and speculative models. The lack of canonical membrane localization signals in the alpha-enolase sequence has suggested the involvement of export routes of non-classically secreted proteins, such as membrane blebbing, membrane flip-flop, endosomal recycling and/or physical association with other proteins that mediate transport to the cell surface^13^. Recently, Zakrzewic and colleagues demonstrated the interaction with caveolae-associated proteins, namely caveolin 1 (Cav-1) and Annexin 2 (Annx2), as well as the functional role of both proteins in the subcellular localization of alpha-enolase and, consequently, in the regulation of cell migration and invasion mediated by surface alpha-enolase^14^.

With the aim of identifying additional signalling pathways and protein partner interactions underlying cell surface localization of alpha-enolase, we applied biochemical and cell biology approaches to three cell lines resembling non-tumourigenic mammary epithelial cells, and low- and high-invasive breast cancer cytotypes. We provide evidence that EGF-signalling upregulates cell surface alpha-enolase, independently of metastatic potential, and identify Hsp70 as a novel interacting partner that favours alpha-enolase localization to the plasma membrane.

## Results

### EGF increases cell migration and up-regulates the expression of alpha-enolase in the cell membrane fraction

The expression of alpha-enolase is elevated on the cell surface of cancer cells, and it has been reported that LPS treatment contributes to protein translocation from the cytoplasm to the plasma membrane and extracellular space^10^. To investigate a possible association between EGF-mediated invasion and the upregulation of alpha-enolase in the membrane compartment, we chose the HB2 mammary epithelial cell line^15^ and the MCF-7 low metastatic breast cancer cell line. Preliminary western blot analyses confirmed the molecular characteristics of the two cell lines, such as different levels of EGF and TLR4 receptors, low/absent expression of vimentin, and a discrete level of alpha-enolase (as shown later in this report), features that made them particularly suitable for a comparative study of effects due to EGF and LPS exposure.

**Figure 1:**
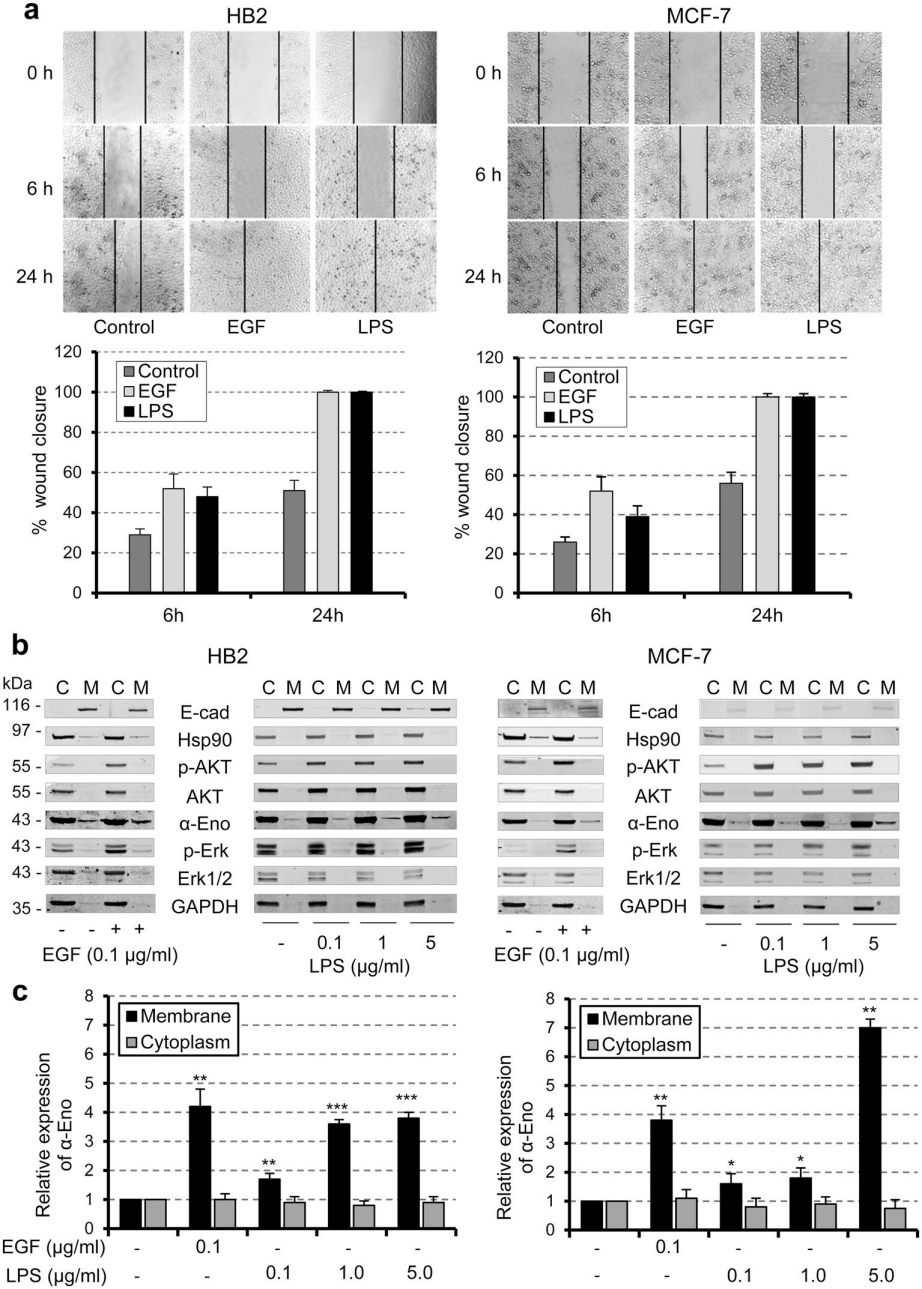
EGF and LPS promote cell migration and the up-regulation of alpha-enolase in the membrane fraction of non-tumourigenic and cancer cells. (**a**) A wound-healing assay was performed to observe changes in the migratory ability of non-tumourigenic HB2 and low metastatic MCF-7 cells after stimulation with 0.1 µg/ml of either EGF or LPS for the indicated length of time. Migration at the edge of the scratch was analysed at 0, 6 and 24 hours, when microscopy images were captured (magnification, 100x, top panel). Cell motility was quantified by measuring the scratch wound, and results are expressed relative to total closure (100%). Each bar represents the mean ± standard deviation of three independent experiments (lower panel). (**b**) HB2 and MCF-7 cells were treated with the indicated EGF or LPS concentrations for 24 hours, after fractionation cell equivalents of cytoplasmic (C) and total membrane (M) fractions were separated by SDS-PAGE and analysed by immunoblotting. E-cadherin (E-cad) and Hsp90 were used to estimate proper fractionation, pAKT and pERK to confirm the effectiveness of treatments. Changes in alpha-enolase (α-Eno) expression levels were analysed in treated versus untreated (−) cells, and GAPDH was used as a further control. (**c**) Quantification of alpha-enolase in cytoplasmic and total membrane fractions. Data were normalised to the densitometric signals of Ponceau staining (see Supplementary Fig. 1) and expressed relative to the untreated control, set at 1. Each data point is the average of three independent experiments, error bars represent standard deviation and p values (*P < 0.05, **P < 0.01, ***P < 0.001) indicate statistical significance.

We firstly analysed the effects of EGF or LPS on cell motility by *in vitro* wound healing assay. As shown in Fig. 1a, the migratory capacity of EGF- or LPS-treated HB2 and MCF-7 cells considerably increased compared to the untreated control cells after 6 h of exposure, and a complete closure was observed in both cases after 24 hours (Fig. 1a). No significant difference in the response of the two cell lines to either EGF or LPS was observed. To investigate a putative correlation between EGFR signalling activation and the expression of alpha-enolase on the cell membrane, we performed a cell fractionation that separated the cytoplasmic fraction and the total membrane fraction. HB2 and MCF-7 cells were treated with EGF or, as a control, with increasing doses of LPS, and then the subcellular fractions were isolated as described in the methods section. Western blot analyses of cytoplasmic Heat shock protein 90 (Hsp90) and plasma membrane E-cadherin were used to estimate proper fractionation, while the expression level of active pAKT and pERK1/2 kinases confirmed the effectiveness of the treatments (Fig. 1b) and filter staining allowed normalization for equal protein loading (supplementary Fig. 1). Following both treatments, the expression of alpha-enolase protein in the membrane subcellular fraction increased compared to the untreated controls, whereas no detectable increase of GAPDH was observed in response to EGF or LPS. Furthermore, we detected a concentration-dependent response to LPS exposure in both cell lines (Fig. 1b,c). The cytoplasmic alpha-enolase levels were unaltered in all conditions (Fig. 1b,c), confirming that the increased expression is not due to an overall rise of alpha-enolase protein in the cell and supporting the finding that EGF, like LPS treatment, selectively modulates alpha-enolase membrane localization.

### EGF increases the localization of alpha-enolase on the plasma membrane, functional consequences on cell invasion

**Figure 2:**
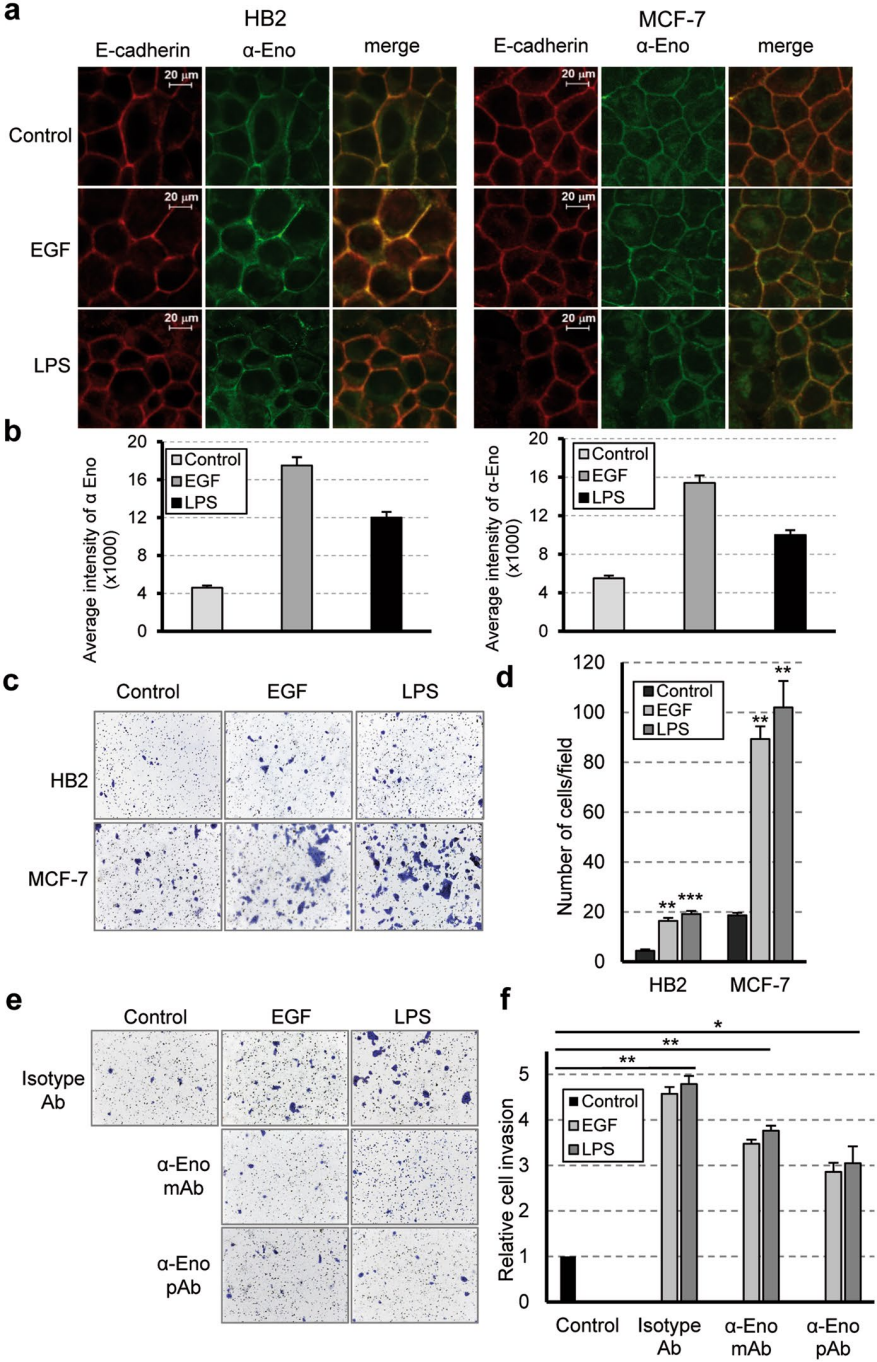
EGF exposure, like LPS treatment, positively affects the cell surface localization of alpha-enolase supporting cell invasion. (**a**) HB2 and MCF-7 cells were treated with 0.1 µg/ml of EGF or 5 µg/ml of LPS for 24 hours, then double-stained with antibodies against alpha-enolase (α-Eno, green, live staining) and E-cadherin (red, staining after cell fixation), and visualized by confocal microscopy. The merged images (right panels) indicate co-localization of the two proteins. (**b**) Fluorescence signals were measured in control and treated cells using the ImageJ image-processing program. Each bar represents the mean ± standard deviation of three independent experiments. (**c**–**f**) Matrigel invasion assays. Unstimulated (Control) and EGF- or LPS- stimulated cells were allowed to invade for 48 hours, (**c**) representative images of cells on the underside of the transwell membrane and (**d**) quantification of cells that invaded. (**e**,**f**) Effect of anti-alpha-enolase antibodies on migration through Matrigel-coated transwell filters. MCF-7 cells were seeded in serum-free medium containing isotype-control antibody (isotype Ab) or anti-alpha-enolase monoclonal (α-Eno mAb, 50 µg/ml) and polyclonal (α-Eno pAb, 15 µg/ml) antibody and incubated in the absence or presence of EGF (0.1 µg/ml) or LPS (5 µg/ml) for 24 hours. (**f**) Quantification of invaded cells is shown relative to the untreated control, set at 1. Results are from three independent experiments, error bars represent standard deviation and p values (*P < 0.05, **P < 0.01, ***P < 0.001) indicate statistical significance.

As previously mentioned, the presence of alpha-enolase on the plasma membrane of monocytes has been well-documented *in vitro* and *in vivo* following inflammatory stimuli^7^; moreover, the association of enolase with the mitochondrial membrane has been reported in yeast and mammalian cardiomyocytes^16–18^. The experimental approach we preliminarily used to evaluate the EGF- or LPS-mediated increase of alpha-enolase in the membrane subcellular fraction did not allow discrimination between the plasma membrane and internal membranes. Thus, we decided to use a live immunostaining approach to monitor cell surface alpha-enolase. Firstly, we identified an antibody, among several commercially available antibodies, that recognizes an epitope of alpha-enolase exposed on the cell surface, and then we developed a protocol for the staining of live cells that allowed the detection of alpha-enolase expression on the cell surface without interference from the highly abundant cytoplasmic counterpart. Immunofluorescence analysis indicated that EGF or LPS treatment increases the amount of alpha-enolase localized in the plasma membrane of both HB2 and MCF-7 cells. The co-localization of alpha-enolase with E-cadherin, a widely used marker of epithelial cells in the plasma membrane, supported the result (Fig. 2a). A computer-assisted image analysis of the immunostained cells indicated that, following EGF or LPS exposure, alpha-enolase levels on the cell membrane were approximately three-times higher when compared to untreated controls (Fig. 2b). To evaluate the impact of enhanced surface alpha-enolase on the invasion capacity of HB2 and MCF-7 cells, a matrigel invasion assay was performed. For both cell lines, the number of cells that migrated in response to EGF or LPS was significantly higher than that for unexposed cells (Fig. 2c,d). In the presence of monoclonal^6, 19^ or polyclonal antibodies against alpha-enolase, the EGF- and LPS-dependent invasiveness of MCF-7 cell was considerably reduced (Fig. 2e). Specific-antibodies addition led to a 25% to 40% reduction in the number of MCF-7 cells passing through the matrigel-coated membrane compared to cell cultured with isotype-control antibodies (Fig. 2f), thus confirming the contribution of surface alpha-enolase to EGF- and LPS-mediated cell invasion.

These results are in line with data reported for other cancer cells, where the stable overexpression or the knocking down of alpha-enolase was shown to promote or inhibit cell invasion, respectively^5, 6, 14, 20–22^, our data are novel for non-tumourigenic mammary epithelial cells.

### Surface alpha-enolase and membrane Hsp70 share a common response to EGF and LPS

**Figure 3:**
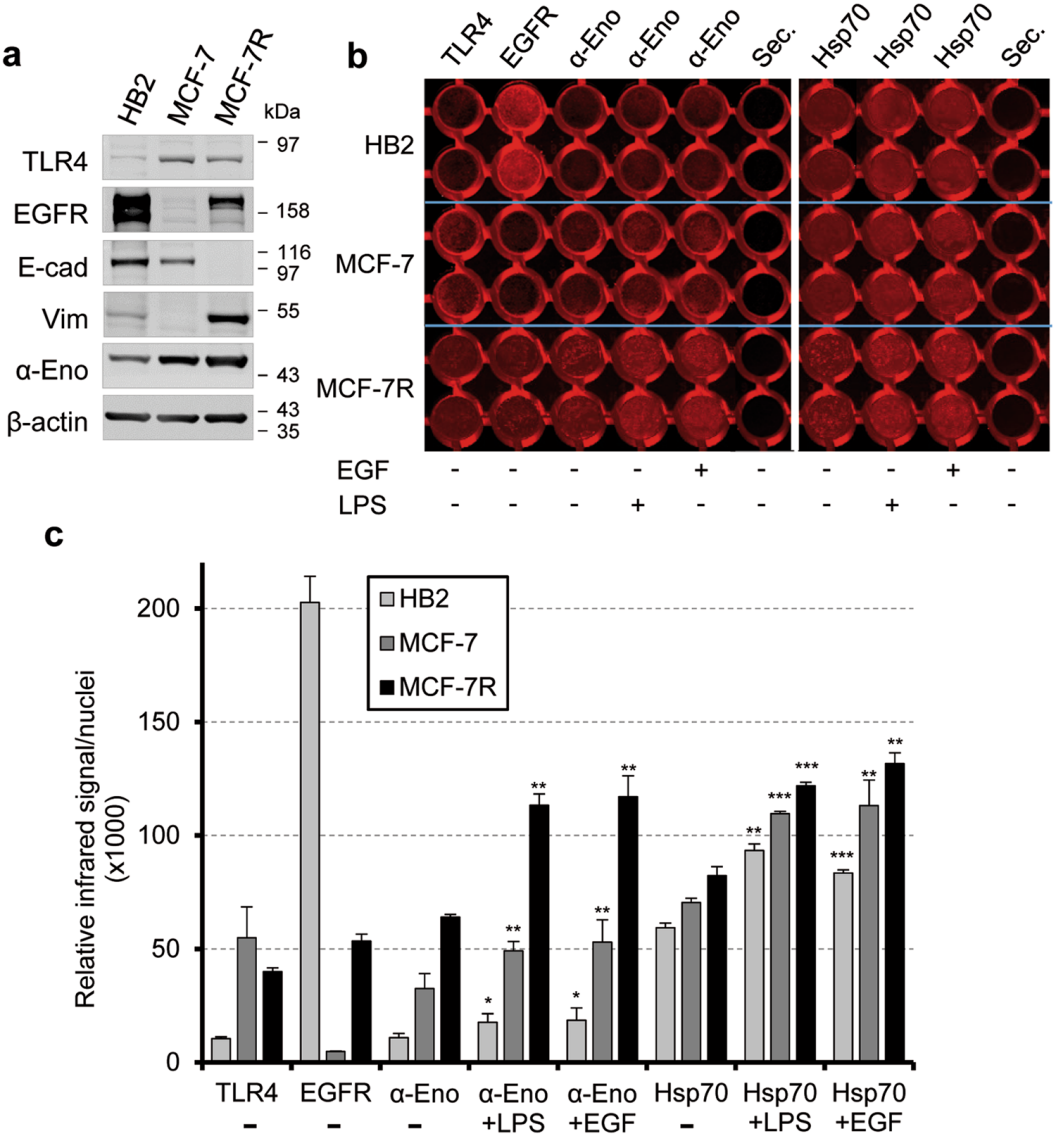
Live on-cell western recapitulates live immunofluorescence staining data. (**a**) Comparative immunoblotting analyses of total proteins from HB2, MCF-7 and MCF-7R cells with antibodies against cell surface receptors, i.e. TLR4 and EGFR, E-cadherin (E-cad), Vimentin (Vim) and alpha-enolase (α-Eno). β-actin is shown as a control of the total proteins loaded per lane. (**b**) Representative image of the infrared signal emission from a 96-well tissue culture plate processed by live on-cell western with the indicated primary antibodies. The image shows ten duplicate wells/cell type of confluent HB2, MCF-7 and MCF-7R cells in control conditions (−) or after treatment (+) with 5 µg/ml LPS or 0.1 µg/ml EGF, for 24 hours. The omission of primary antibodies (sec.) is shown as a control of specificity. (**c**) The bar graph shows the relative mean infrared intensity for live on-cell western analyses, corrected for nuclei staining. Each data point is the average of three independent experiments, error bars represent standard deviation and p values (*P < 0.05, **P < 0.01, ***P < 0.001) indicate statistical significance relative to the untreated control.

To further explore the effect of EGF and LPS treatments on alpha-enolase localization to the plasma membrane, we developed a live on-cell western technique that quantitatively measures plasma membrane alpha-enolase by imaging living cells. We performed such analysis on HB2, MCF-7 and the doxorubicin-resistant counterpart, MCF-7R. All three cell lines express EGFR and TLR4 receptors, although at different levels; notably, MCF-7R has lost E-cadherin expression and acquired Vimentin, while showing higher levels of EGFR and alpha-enolase (Fig. 3a), all features of cancer cell aggressive phenotype. Matrigel invasion assay confirmed the increased invasive capacity of MCF-7R cells compared to the parental MCF-7 cells (supplementary Fig. 2), behaviour which is consistent with the acquisition of doxorubicin resistance^23^.

Antibodies directed against extracellular epitopes of TLR4, EGFR and alpha-enolase, respectively, were used to set up experimental conditions for assessing the localization of the selected proteins on the cell surface of live cells by the on-cell western approach.

Figure 3b shows the red-scale infrared signal at 700 nm measured using an infrared imaging system from a 96-well tissue culture plate; the fluorescent signals were quantified on a fluorescent scanner, normalizing to the approximate number of cells, measured by nuclear staining. As expected, the analysis indicated that all three cell lines expressed TLR4 and EGFR on their surface, and the relative expression in each cell line was consistent with the overall expression level detected in total cell lysates (compare Fig. 3a and c). Alpha-enolase was expressed on the surface of the three cell lines, and its amount correlated with the tumourogeneity and invasiveness of cells, as the doxorubicin-resistant MCF-7R showed the highest steady-state level of expression (Fig. 3c).

Next, we performed EGF and LPS treatments and measured the expression of surface alpha-enolase; we also evaluated the levels of plasma membrane Hsp70, another multifunctional protein detected on the cell surface of human and mouse tumours, using an antibody specifically recognizing membrane-bound Hsp70^24^. The relative mean infrared signal intensities indicated that all three cell lines respond to EGF and LPS exposure by upregulating alpha-enolase on their surface, and, surprisingly, we also detected an increased expression of membrane Hsp70 (Fig. 3b and c). The on-cell western blot recapitulated and corroborated what had been previously observed by subcellular fractionation and immunofluorescence analysis (Figs 1 and 2) and, as a novel finding, suggested that the trafficking of the intracellular chaperone Hsp70 to the cell membrane may share a common pathway with alpha-enolase.

### Alpha-enolase interacts with Hsp70

**Figure 4:**
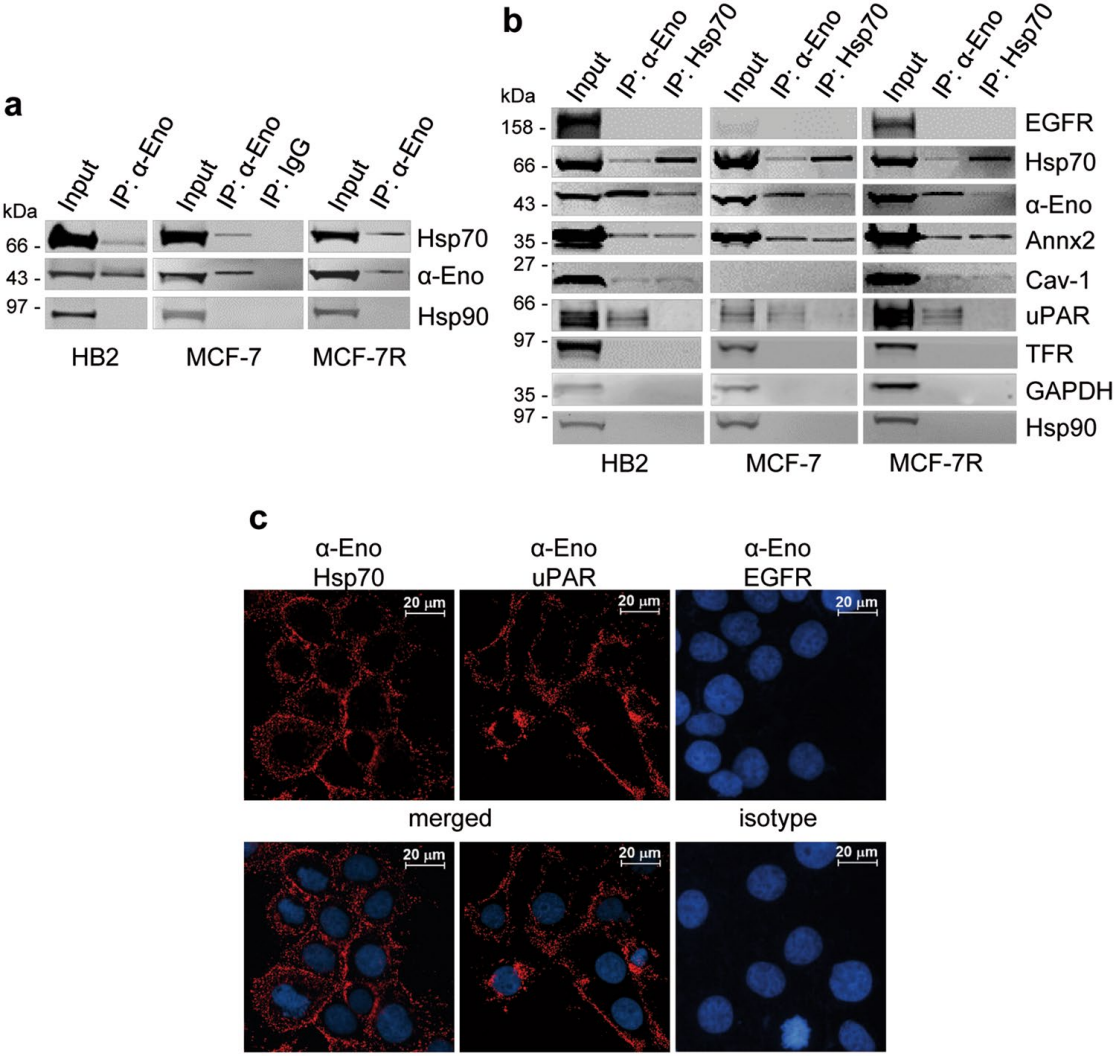
Interaction between Hsp70 and alpha-enolase. (**a**) Association of endogenous Hsp70 and alpha-enolase proteins in the cytoplasmic fraction of HB2, MCF-7 and MCF-7R cells. Cytoplasmic extracts were immunoprecipitated with anti-alpha-enolase monoclonal antibody (IP: α-Eno) or a pre-immune isotype-matched antibody (IP: IgG), then analysed by immunoblotting using anti-Hsp70 polyclonal antibody, anti-alpha-enolase polyclonal and anti-Hsp90 monoclonal antibody. Input represents 4% of the cell extract used for each IP sample. Hsp70 was specifically co-immunoprecipitated with alpha-enolase in the three cell lines. (**b**) Association of endogenous Hsp70 and alpha-enolase proteins in the plasma membrane fraction of HB2, MCF-7 and MCF-7R cells. Plasma membrane fractions were immunoprecipitated with anti-alpha-enolase monoclonal antibody (IP: α-Eno) or anti-Hsp70 monoclonal antibody (IP: Hsp70). Immunoprecipitated complexes were analysed by immunoblotting for the presence of the indicated proteins. Annx2, Cav-1 and uPAR were used as positive controls for alpha-enolase interactions. No co-precipitation of EGFR, TFR, GAPDH or Hsp90 was observed with either Hsp70 or alpha-enolase, while Annx2 and Cav-1 were specifically co-immunoprecipitated with both proteins. Note that Cav-1 expression is undetectable in the MCF-7 plasma membrane fraction compared to the other cell lines. Hsp70 was specifically co-immunoprecipitated with alpha-enolase, and, similarly, alpha-enolase was specifically co-immunoprecipitated with Hsp70. Input represents 6% of the cell extract used for each IP sample. (**c**) Representative images of PLA assay detecting the proximity of surface-localized alpha-enolase and Hsp70. HB2 cells were live-stained with alpha-enolase-specific antibody together with anti-Hsp70 (α-Eno, Hsp70), anti-uPAR (α-Eno, uPAR), anti-EGFR (α-Eno, EGFR) and nuclei counterstained with DAPI (blue). A positive amplified signal (red) indicated that alpha-enolase and either Hsp70 or uPAR (positive control) were co-located within 40 nm, while no signal was obtained after incubation with either anti-EGFR or isotype-matched antibody (right panels). Results are representative of three independent experiments with comparable outcomes.

Cell surface alpha-enolase and Hsp70 showed a common response to EGF or LPS exposure by on-cell western of living cells. This observation prompted us to investigate whether the two proteins may interact. To test the putative interaction, cytoplasmic HB2, MCF-7 and MCF-7R cell extracts were immunoprecipitated with anti-alpha-enolase antibody, followed by immunoblotting with anti-Hsp70 antibody. Figure 4a shows that alpha-enolase co-precipitated with Hsp70 but not with Hsp90, another abundant chaperone, indicating that the endogenous alpha-enolase and Hsp70 proteins may interact in the cytoplasmic compartment and that alpha-enolase is not a classical Hsp90 “client” protein^25^.

The protein-protein interaction analysis was further extended to the cell surface compartment; native plasma membrane fractions were isolated from HB2, MCF-7 and MCF-7R cells and immunoprecipitated with anti-alpha-enolase or anti-Hsp70 antibodies, then the precipitated complexes were probed with antibodies against a selected group of proteins. No co-precipitation of EGFR or TFR receptor, GAPDH or Hsp90 protein was detected, neither with endogenous alpha-enolase nor with Hsp70 (Fig. 4b). Conversely, the newly identified interaction between alpha-enolase and Hsp70 and the previously reported interactions of alpha-enolase with Annx2, Cav-1 and urokinase plasminogen activator receptor (uPAR)^5, 14^ were confirmed in the plasma membrane subcellular fraction. Interestingly, the co-immunoprecipitation results indicated the association of Hsp70 with the alpha-enolase interactors, namely Annx2 and Cav-1, but not with uPAR (Fig. 4b), suggesting either the presence of a plasma membrane multiprotein complex and/or a route of translocation to the cell surface common to alpha-enolase and Hsp70.

Next, we performed an *in situ* Proximity Ligation Assay (PLA) in live cells to further examine the nature of the interaction between alpha-enolase and Hsp70 on the cell surface. The detection of a positively amplified signal by this method indicates that two selected proteins are located within 40 nm of each other, thus visualizing protein localization in close proximity or co-localization. Figure 4c shows that alpha-enolase was found to co-localize with Hsp70 in the plasma membrane and, as previously reported by Hsiao and colleagues, with uPAR^5^, while no positively amplified signal was detected using antibodies against EGFR or isotype-matched controls. Consistently with the co-immunoprecipitation data, the PLA results supported by other means that alpha-enolase and Hsp70 interact with each other on the cell surface.

### The surface expression of alpha-enolase is downregulated by Hsp70 knockdown

**Figure 5:**
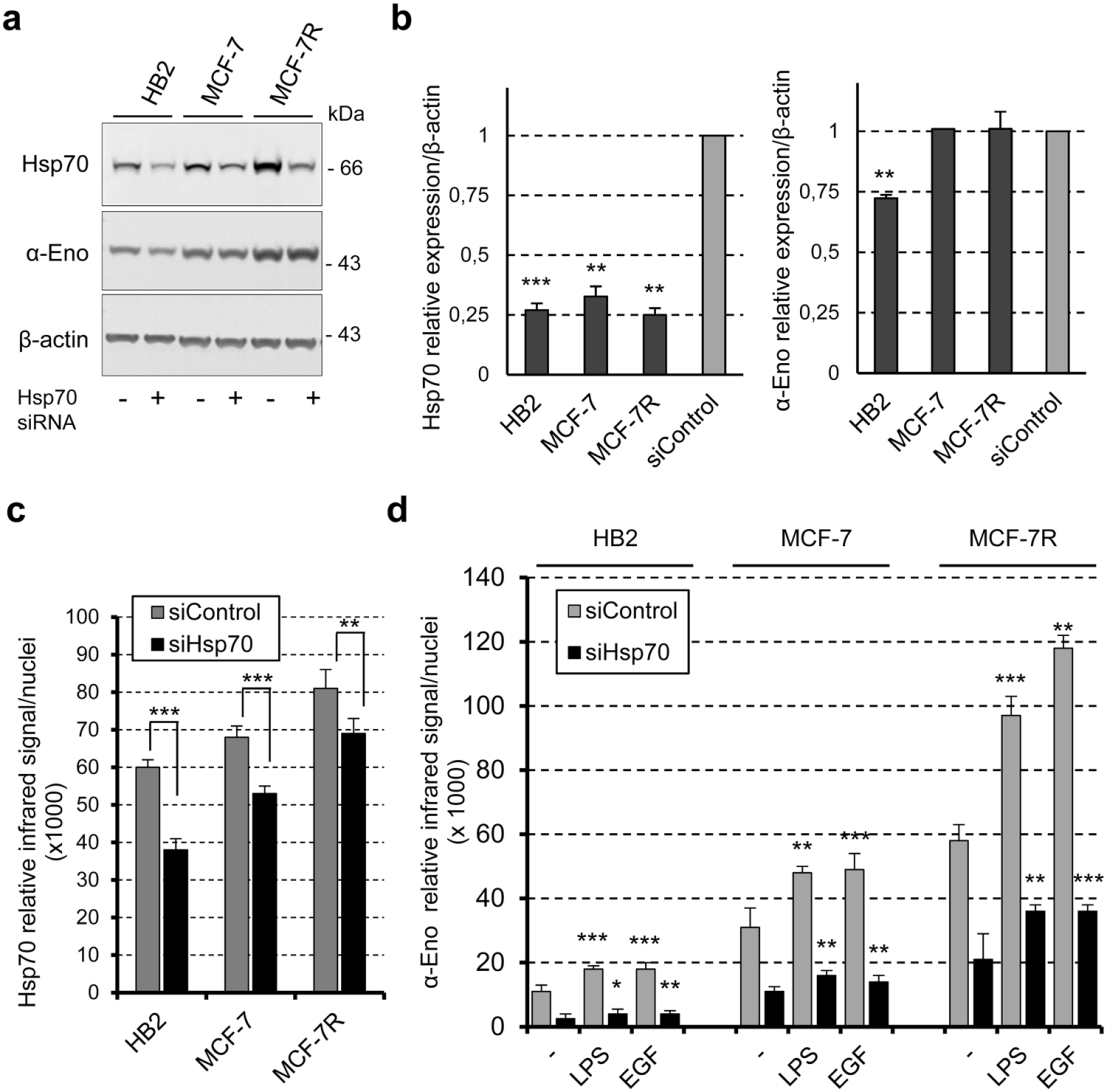
The knockdown of Hsp70 negatively affects surface-localized alpha-enolase. (**a**) HB2, MCF-7 and MCF-7R cells were treated with Hsp70-specific siRNA (+) or unrelated siRNA as a control (−), and immunoblotting analysis of total lysates with anti-Hsp70 and anti-alpha-enolase (α-Eno) was performed. β-actin is shown as a control of the total proteins loaded per lane. (**b**) Quantification of Hsp70 and alpha-enolase in Hsp70-silenced cells. Data were normalised to the densitometric signals of β-actin and expressed relative to the level detected in cells treated with unrelated siRNA (siControl), set at 1. (**c**,**d**) Live on-cell western of Hsp70-silenced cells. The bar graph in (**c**) shows the relative mean infrared intensity for membrane Hsp70, corrected for nuclei staining. Transiently-silenced cells were either untreated or treated with 5 µg/ml LPS or 0.1 µg/ml EGF for 24 hours, and then analysed for the expression of surface-localized alpha-enolase by on-cell western (**d**). Results are the average of three independent experiments, error bars represent standard deviation and p values (*P < 0.05, **P < 0.01, ***P < 0.001) indicate statistical significance relative to the untreated control.

To explore the functional role of Hsp70 in the subcellular localization of alpha-enolase, we silenced Hsp70 by siRNA transfection in HB2, MCF-7 and MCF-7R cells. Firstly, Hsp70 expression was monitored in total cell extracts to assess the extent of silencing; we detected about 70% of expression reduction in all three cell lines (Fig. 5a,b). Hsp70 knockdown did not influence total alpha-enolase expression in MCF-7 and MCF-7R cells, and to some extent affected total level (about 30% reduction) in non-tumourigenic HB2 cells, which express less alpha-enolase (Fig. 5a,b). Conversely, in all three cell lines the on-cell western analyses showed a substantial and comparable downregulation of membrane Hsp70 and, to a larger extent, of surface-localized alpha-enolase in silenced cells with respect to controls treated with unrelated siRNA (Fig. 5c,d).

Finally, to further investigate the involvement of Hsp70 in the localization of alpha-enolase to the plasma membrane, the effects of Hsp70 silencing on EGF and LPS treatments were explored in HB2, MCF-7 and MCF-7R cells by the on-cell western approach. Figure 5d shows how the knockdown of Hsp70 counteracted the EGF- and LPS-mediated upregulation of alpha-enolase in the plasma membrane compartment, drastically reducing surface alpha-enolase compared to the control cells. The effect of Hsp70 knockdown is specific for surface alpha-enolase, since the total level of alpha-enolase was not affected in breast cancer cells. Collectively, these data strongly supported a functional role of the interacting protein Hsp70 in regulating alpha-enolase surface localization.

## Discussion

Elevated levels of alpha-enolase expression have been reported for many cancer types, and alterations of cytosolic enzyme levels have been widely considered a biomarker of malignancy^26, 27^. In addition to its major role in glycolysis, the alpha-enolase protein performs multiple functions, depending on its subcellular localization: a nuclear short variant, Myc promoter-binding protein 1 (MBP-1), acts as a transcriptional repressor, while the cytosolic protein, when translocated to the cell surface, binds to plasminogen, contributing to pericellular plasmin production and cell motility. So far, no other roles have been elucidated for the protein exposed on the cell membrane, while an additional function, still poorly investigated, is likely to be exerted by the alpha-enolase secreted in extracellular vesicles; notably, alpha-enolase is reported to be among the most abundant proteins in exosomes^28^.

The nuclear short form, MBP-1 and the protein localized to cell surface have attracted attention from researchers for their involvement in tumour growth, invasion and metastasis development^29, 30^. A role for MBP-1 in down-regulating tumour invasion and metastasis has been proposed for gastric cancer using mouse xenograft models^31^, and MBP-1 expression has been positively correlated to patient outcomes in primary invasive ductal carcinoma of the breast^32^. More recently, antibodies targeting surface-localized alpha-enolase have been shown to inhibit the metastatic spreading of lung cancer cells and pancreatic adenocarcinoma cells in immunosuppressed mice^5, 6^. Proteomics-based studies on purified plasma membranes have confirmed the surface localization of alpha-enolase in lung, pancreatic and breast cancer cells^33–35^. A few studies in yeast and mammalian cells have indicated the presence of enolase in discrete membrane regions, i.e. membrane rafts^36, 37^. Moreover, Wygrecka’s group has demonstrated the interaction of alpha-enolase with Cav-1 and Annx2 in the highly invasive MDA-MB-231 cell line and proposed that the altered levels of intracellular Ca2+ play a pivotal role in the regulation of translocation from the cytosolic compartment to the plasma membrane^10, 14^.

In this study, we have focused on surface-localized alpha-enolase, adding novel insights to the stimuli and interactors contributing to its cell surface expression, which ultimately might be the prerequisite for a further release of the protein into the extracellular space.

Here, we have demonstrated for the first time that EGF signalling is involved in the up-regulation of surface-localized alpha-enolase both in non-tumourigenic mammary epithelial cells and in breast cancer cells, independently from the steady-state level of surface expression and the metastatic potential of each cell type. Our data also confirm previously studies identifying LPS exposure among the stimuli that upregulate cell surface alpha-enolase in monocytoid cells and breast cancer cells^7, 10^, in accordance to the presence of TLR4 receptors in both cell types^9, 38, 39^. The results are in agreement with the notion that stimuli promoting tumour progression, as demonstrated for TGF-α, TNF-α and CCL2 in MDA-MB-231 cells^10^, trigger surface alpha-enolase expression.

A further novel finding of this study concerns the interaction of surface-localized alpha-enolase with another multifunctional protein, Hsp70, the major stress-inducible member of the Hsp70 family. This protein, like alpha-enolase, does not present a consensual secretory signal; nevertheless, a lot more is known about membrane-bound Hsp70, which translocates to the plasma membrane after stress and is released into the extracellular environment in a membrane-associated form that is capable of activating immune cells^40^. In tumour cells, but not in non-malignantly transformed cells, Hsp70 appears to be stably associated with the plasma membrane^41^.

We have shown for the first time that Hsp70, like alpha-enolase, in response to EGF and LPS stimuli, is overexpressed in the plasma membrane where the two proteins co-localize and interact, likely in discrete membrane regions. As an additional insight towards the understanding of the molecular mechanisms underlying the localization of alpha-enolase to the cell membrane, we propose a functional involvement of Hsp70, which may contribute to alpha-enolase transport and/or stabilization on the plasma membrane. This hypothesis is strongly supported by the fact that the two proteins interact in the cytoplasm and the plasma membrane subcellular compartments, and that silencing Hsp70 expression resulted in a specific downregulation of the surface alpha-enolase steady-state level, as well as in the inhibition of EGF- and LPS-mediated effects.

More in-depth studies are needed to fully dissect the signalling pathways and the molecular mechanism(s) leading to the cell surface exposure of alpha-enolase and to clarify the existence of specific membrane subdomains, where enolase and its interactors may contribute to multiple functions in addition to plasminogen binding, both in non-tumourigenic and cancer cells.

## Methods

### Reagents

LPS and EGF were purchased from Sigma-Aldrich, n-Octyl-D-glucoside was purchased from Santa Cruz Biotechnology. Primary antibodies were: mouse monoclonals Eno 276/3 and 72/1 against alpha-enolase^19^; mouse monoclonal cmHsp70.1 against membrane Hsp70^24^; goat anti-alpha-enolase, rabbit anti-E-cadherin, mouse anti-Hsp90, rabbit anti-Hsp70,﻿﻿ goat anti-GAPDH, rabbit and mouse anti-EGFR, mouse anti-TFR, rabbit anti-uPAR, rabbit anti-Vimentin, goat anti-Lamin B (Santa Cruz Biotechnology); rabbit anti-phospho-Akt, mouse anti-Akt, rabbit anti-Phospho-p44/42 MAPK (Erk1/2) (Cell Signaling Technologies); mouse anti-ERK1/2 (Proteintech Group), mouse anti-β-actin (Sigma-Aldrich); rabbit anti-TLR4 (Novus Biologicals); rabbit anti-Caveolin-1 (Upstate Biotechnology); mouse anti-Annexin-A2 (BD Biosciences). Alexa Fluor-conjugated secondary antibodies were purchased from ThermoFisher Scientific Inc.

### Cell culture

The human MCF-7 breast cancer cell line was purchased from American Type Culture Collection (ATCC, Rockville, MD, USA); the doxorubicin-resistant counterpart, MCF-7R^42^, and the human HB2 non-tumourigenic mammary epithelial cell line^15^ were kindly provided by Natale D’Alessandro and Ida Pucci-Minafra (University of Palermo, Italy), respectively. MCF-7 and MCF-7R cells were cultured in Dulbecco’s modified Eagle medium (DMEM) supplemented with 10% heat-inactivated fetal bovine serum (FBS), glutamine (4 mM) and penicillin/streptomycin (100 μg/ml). For HB2 cells, culture medium (DMEM low-glucose) was supplemented with hydrocortisone (5 μg/ml) and bovine insulin (10 μg/ml).

For treatment with EGF (0.1 µg/ml) and LPS (0.1, 1 or 5 µg/ml), cells were plated at a density of 2 × 10^4^/cm^2^, cultured in complete culture medium for 24 hours, then serum-starved overnight. Cells were stimulated in serum-free medium supplemented with the indicated quantity of ligands for an additional 24 hours.

### Wound-healing assay

HB2 and MCF-7 cells were grown near to confluence in a 6-well plate and serum-starved overnight. A scratch was made on the cell monolayer using a sterile pipette tip, and cells were treated with either EGF or LPS (0.1 µg/ml) or left untreated in fresh serum-free medium for 24 hours. Pictures were captured by a Leica DM IL LED microscope at 0, 6 and 24 hours after scratching. The rate of wound closure was assessed by measuring the distance between the migrating cell boundaries. All experiments were performed in triplicate.

### Cell Invasion Assay

For cell invasion assay, serum-free medium (750 μl) with or without EGF (0.1 µg/ml) or LPS (5 µg/ml) was added into the lower chambers of a 24 transwell plate, 2 × 10^4^ cells were seeded into the upper chamber coated with matrigel (8.0-μm pore size, Corning). After 48 hours of incubation, non-migrating cells were removed with a cotton swab and cells that had migrated at the bottom of the membrane were fixed with ice cold acetone/methanol (1:1) and stained with crystal violet (0.5% in 20% methanol) for 1 h. The cells located on the underside of the filter were photographed by phase contrast microscopy (x100 magnification) and counted (5 fields/filter). The experiments were performed in triplicate. Where indicated, mouse anti-alpha enolase 72/1 (50 µg/ml) and goat anti-alpha-enolase (15 µg/ml) or isotype-matched control antibody were added to the appropriate inserts for 24 hours; thereafter, cells were processed as described.

### Cell extracts and immunoblotting

Total protein extracts were prepared as previously described^43^, cytoplasmic and total membrane fractions were isolated using the Calbiochem ProteoExtract Subcellular Proteome Extraction Kit, and native plasma membranes were obtained with the BioVision Membrane Protein Extraction Kit, according to the manufacturers’ instructions. All buffers were supplemented with protease and phosphatase inhibitor cocktails (Sigma-Aldrich), and protein concentrations were determined by the Bradford protein assay (Bio-Rad Laboratories).

Total proteins or cytoplasmic extracts (20–30 μg) and corresponding total membrane cell equivalents were separated on 10% polyacrylamide gels (SDS-PAGE) or NuPage Novex 4–12% Bis-Tris precast gels (ThermoFisher Scientific Inc.), and then transferred onto a nitrocellulose membrane. Primary antibodies were revealed with secondary antibodies, either conjugated to IRDye® 800CW (LI-COR) or Alexa Fluor 680, using the Odyssey infrared imaging system (LI-COR Biosciences) according to the manufacturer’s instructions.

Normalization for total proteins was based on β-actin signals, whereas the densitometric signals of reversible Ponceau S staining were used to normalize cytoplasmic and total membrane fractions^44^.

### Co-immunoprecipitation

For the co-immunoprecipitation of plasma membrane proteins, the final membrane pellet from the Biovision Kit was re-suspended in Phosphate-buffered saline (PBS) containing 1% Triton X-100, 60 mM n-Octyl-D-glucoside, 2 mM EDTA, and supplemented with protease and phoshatase inhibitors (Sigma-Aldrich). Plasma membrane proteins (180 μg) or the corresponding cytoplasmic extract (500 μg) were incubated overnight with mouse monoclonal antibodies (10 μg), either against alpha-enolase (Eno 276/3) or surface Hsp70 (cmHsp70.1), which had been previously cross-linked to agarose beads using the Cross-linking Immunoprecipitation Kit from ThermoFisher Scientific. The specificity of the precipitated immunocomplexes was assessed using IgG isotype controls. The immunoprecipitated proteins were eluted off the beads, incubated in a low pH buffer for 5 minutes at room temperature, separated on a 4–12% Bis-Tris Plus Gel (ThermoFisher Scientific Inc.) and analysed by immunoblotting.

### Immunofluorescence microscopy

Cells were plated at a density of 1.8 × 10^4^ cells/well in LAB-TEK Chamber Slide, 8well, and cultured for 24 hours. The next day, cells were serum-starved overnight and then treated with either EGF or LPS or left untreated for 24 hours. For live-cell immunofluorescence, cells were incubated with goat anti-alpha-enolase antibodies (4 µg/ml) in DMEM supplemented with 5 mM NaN_3_, to minimize capping and/or recycling of surface proteins, for 30 min at room temperature. After three washings in ice-cold PBS plus NaN_3_ (5 mM) on ice, cells were fixed in 4% paraformaldehyde for 15 min at room temperature. Fixed cells were blocked with 1% BSA in PBS for 30 min, and then incubated with rabbit anti-E-cadherin (4 µg/ml) in PBS-1% BSA overnight at 4 °C. AlexaFluor 488-conjugated Donkey anti-goat IgG and AlexaFluor 594-conjugated Goat anti-rabbit IgG were used as secondary antibodies at a dilution of 1:500 for 1 hour at room temperature. DNA was counterstained with 4′6-diamidino-2-phenylindole (DAPI) using Vectashield (Vector Laboratories). Primary-antibody omission demonstrated the specificity of the immunostaining. Images were captured using an Axioskop 2 plus (Carl Zeiss) microscope equipped with a 40x objective or a FluoView FV10i confocal laser-scanning microscope (Olympus) equipped with a 60x oil objective. The fluorescent staining intensity of alpha-enolase was quantified using the ImageJ software on at least five representative fields, and data are presented as means ± standard deviation. All experiments were performed in triplicate.

### Live staining by on-cell western

The on-cell western protocol was conducted according to Milovancev *et al*.^45^, with some modifications. Cells were plated at a density of 0.7 × 10^4^ cells/well in a 96-well plate and cultured for 24 hours. After serum-starving overnight, cells were either left untreated or treated with EGF (0.1 µg/ml) or LPS (5 µg/ml) in serum-free medium for 24 hours. Live-staining for surface alpha-enolase and surface Hsp70 was performed as previously described in the immunofluorescence microscopy section using goat anti-alpha-enolase and mouse monoclonal cmHsp70.1 (15 μg/ml), respectively. A mouse monoclonal antibody against EGFR that recognizes a cell surface epitope and a rabbit anti-TLR4 binding to the extracellular domain of the receptor were used similarly on live cells. The use of antibodies against an intracellular antigen, laminin B, and the omission of primary antibody confirmed the specificity of the cell surface staining. After incubation with primary antibodies, cells were fixed in 4% paraformaldehyde, blocked with 1% BSA in PBS for 30 min, then incubated with AlexaFluor 680-conjugated secondary antibody (1:2000) in PBS-1% BSA for 1 hour at room temperature. Cell were then washed extensively and imaged on a LI-COR scanner. Nuclei were counterstained with Hoechst 33258 (Sigma-Aldrich) and the fluorescent signal was detected using a fluorescent plate reader (BioRad Laboratories). The fluorescent signal from the secondary antibodies was subtracted of blank average signal, and then normalized against the Hoechst staining, to account for differences in the number of cells. Each condition had three technical replicates, and data are presented as means ± standard deviation of at least three experiments.

### Proximity Ligation Assay (PLA)

The Duolink *in situ* PLA (Sigma-Aldrich) was performed following the manufacturer’s protocol, with minor modifications. Briefly, cells were cultured and live-stained as they were for the immunofluorescence and on-cell western procedures. Cells were incubated in DMEM/5 mM NaN_3_ with goat anti-alpha-enolase plus mouse anti surface Hsp70, or goat anti-alpha-enolase plus rabbit anti-uPAR as a positive control. All antibodies were used at a concentration of 15 μg/ml. Live-staining with goat anti-alpha-enolase plus mouse anti-EGFR was performed as a negative control. Omission of antibodies or incubation with isotype-matched pre-immune antibodies further demonstrated the specificity of the PLA reaction. The next steps, such as incubation with specific secondary antibodies and a connector oligonucleotide, enzymatic DNA ligation in the PLA ligation buffer, and polymerase amplification of the ligation products, were performed as recommended. Finally, fluorescence-labeled oligonucleotides complementary to the sequence on the circle were added, and nuclei were counterstained with DAPI. Fluorescent signals were visualized using a FluoView FV10i confocal laser-scanning microscope (Olympus) equipped with a 60x oil objective.

### Small interfering RNA (siRNA) transfection

HB2, MCF-7 and MCF-7R cells (2 × 10^5^) were reverse transfected with human Hsp70 siRNA (Santa Cruz) or control siRNA (AllStars, Qiagen) at a final concentration of 30 nM, using Lipofectamine RNAiMAX transfection reagent according to the manufacturer’s instructions (ThermoFisher Scientific Inc.). Cells were processed for immunoblotting and on-cell western 48 hours after transfection.

### Statistical analysis

All data are presented as means ± standard deviation of at least three experiments. The statistical significance of differences following EGF and LPS treatment was assessed by paired, one-sided Student’s T-Test; statistical differences are presented at probability levels of *P < 0.05, **P < 0.01, ***P < 0.001.

## Electronic supplementary material

Supplementary figures
