# Supplementary material for: Pro-invasive stimuli and the interacting protein Hsp70 favour the route of alpha-enolase to the cell surface
Source: Sci Rep. 2017 Jun 19;7:3841. doi: 10.1038/s41598-017-04185-8 (PMC5476664; doi:10.1038/s41598-017-04185-8)
Supplement: Supplementary file 1 — Supplementary figures [file 41598_2017_4185_MOESM1_ESM.pdf]

Pro-invasive stimuli and the interacting protein Hsp70 favour the route of alpha-enolase to the cell surface

Giovanni Perconti[a,1], Cristina Maranto[a,1], Daniele P. Romancino[a], Patrizia Rubino[a], Salvatore Feo[a,b], Antonella Bongiovanni[a], and Agata Giallongo[a,*]

a. Institute of Biomedicine and Molecular Immunology "A. Monroy" (IBIM), National Research Council (CNR), Palermo, Italy

b. Department of Biological, Chemical and Pharmaceutical Sciences and Technologies (STEBICEF), University of Palermo, Italy

Supplementary information

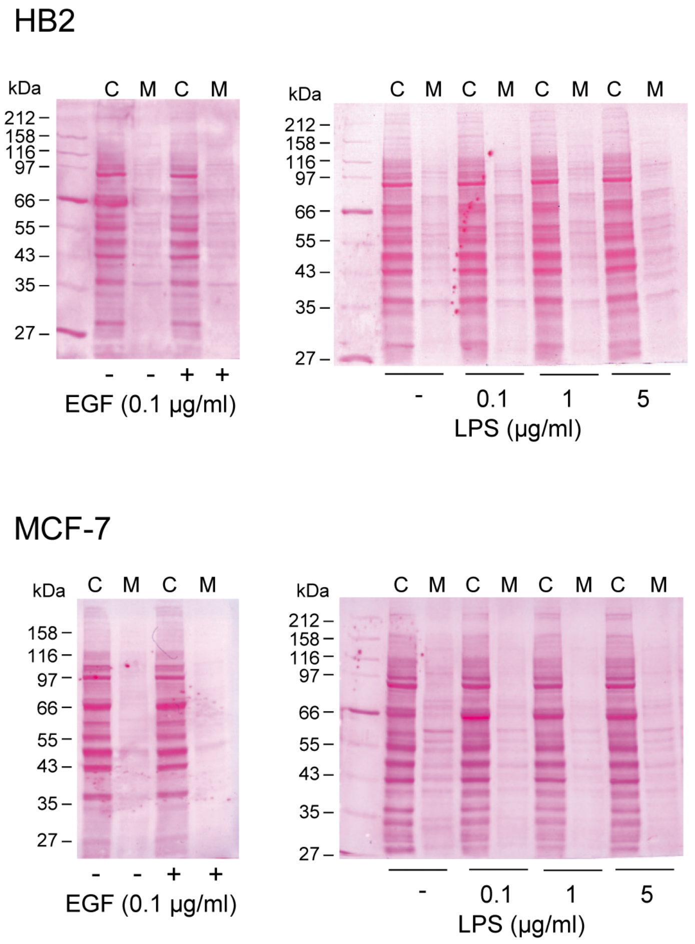

Supplementary Figure-1 (Giallongo)

Ponceau S stained nitrocellulose membranes containing the transferred cytoplasmic (C) and total membrane (M) fractions of HB2 and MCF-7 shown in figure **1 b.**

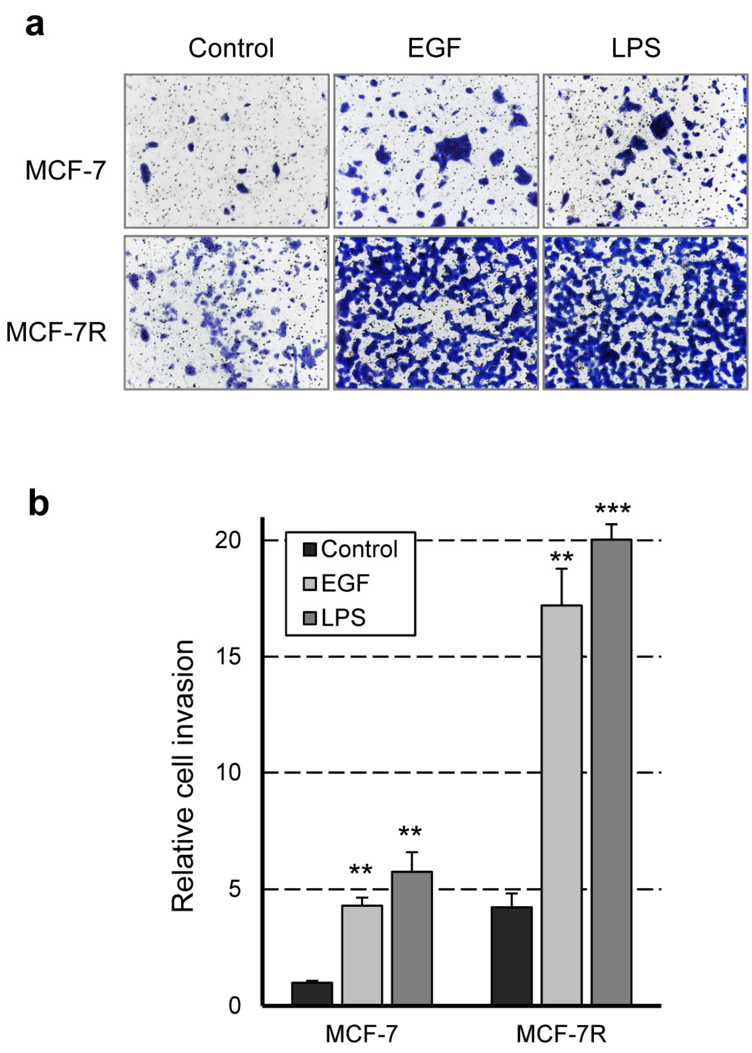

Supplementary Figure-2 (Giallongo)

Matrigel invasion assays. **(a)** MCF-7 and the doxorubicin-resistant MCF-7R cells were either untreated (Control) or treated with EGF (0.1 µg/ml) or LPS (5 µg/ml) and allowed to invade for 48 hours. **(b)** Quantification of invaded cells is shown relative to the untreated MCF-7 cells, set at 1. Results are from three independent experiments, error bars represent standard deviation and p values (** P<0.01, *** P<0.001) indicate statistical significance.
